# Analysis of the Compression Behaviour of Reinforced Photocurable Materials Used in Additive Manufacturing Processes Based on a Mask Image Projection System

**DOI:** 10.3390/ma14164605

**Published:** 2021-08-16

**Authors:** Jordi Bonada, Pol Barcelona, Miquel Casafont, Josep Maria Pons, Jose Antonio Padilla, Elena Xuriguera

**Affiliations:** 1 Department of Strength of Materials and Structural Engineering, ETSEIB, Universitat Politècnica de Catalunya, Avda. Diagonal 647, 08028 Barcelona, Spain; miquel.casafont@upc.edu (M.C.); josep.maria.pons@upc.edu (J.M.P.); 2 Department of Material Science and Physical Chemistry, Universitat de Barcelona, c/Martí i Franquès 1, 08028 Barcelona, Spain; pbarcelona94@ub.edu (P.B.); japadilla@ub.edu (J.A.P.); xuriguera@ub.edu (E.X.)

**Keywords:** additive manufacturing, mask image projection, mechanical properties, photocurable materials, reinforced materials

## Abstract

Mask image projection based on stereolithography is an additive manufactured technology based on a Frontal Photopolymerization Process. Therefore, photocurable resins are used to build-up parts layer by layer. In this paper, alumina particles have been used as a reinforcement filler in order to improve the material stress-strain behaviour. In addition, the increment of the photoconversion ratio is a key factor to enhance the mechanical properties. Consequently, a numerical model has been used to determine the optimal printing parameters to enhance the elastic mechanical properties of printed parts according to the characteristics of photocurable materials. Stable and homogeneous reinforced materials have been obtained with an alumina content ranging from 5 to 15 wt%. Furthermore, the compression behaviour of reinforced materials has been analysed by means of experimental tests. The results show an enhancement of mechanical properties after the addition of reinforcement fillers, obtaining a maximum improvement in 10 wt% of solid load content. Finally, the influence of the sample’s orientation on the construction platform has been discussed.

## 1. Introduction

Mask image projection based on stereolithography (MIP-SL), also known as digital light processing (DLP), is an additive manufacturing technology which uses a frontal photopolymerization process (FPP) to manufacture each material layer. This technology employs a digital micro device (DMD) to emit a controlled light energy dose on a photocurable resin to built-up the final part layer by layer [1,2]. MIP-SL process can have a higher manufacturing speed than stereolithography (SLA) due to the simultaneous energy delivery in each layer. Furthermore, both FPP technologies have a high resolution, which is one of their main characteristics.

One of the main disadvantages of the MIP-SL process is the lower mechanical properties of photocurable materials. Actually, the values of some mechanical properties, such as elastic modulus or tensile strength, depend on the material conversion ratio achieved during the printing process or after a post-curing treatment [3,4]. Furthermore, a non-uniform conversion ratio distribution could cause some non-desired effects, such as non-uniform material properties in the printed parts. Therefore, research has recently been done to develop numerical models based on FPP to determine the conversion ratio distribution according to main material and printing parameters [3,5,6,7]. Consequently, optimized printing parameters can be calculated in order to enhance the mechanical properties of printed parts.

On the other hand, the use of reinforcement particles or fibres in photocurable materials has become an interesting approach to increase some mechanical properties of samples obtained by means of FPP technologies [8,9,10]. An enhancement of elastic modulus is achieved after the addition of particles to base or commercial photocurable resins. The use of nanometric particles as reinforcement for polymers improves the mechanical properties more efficiently than the use of larger particles, reaching higher solid loads without reaching a limit where the properties go down again [11]. On the contrary, the addition of reinforcement has a relevant influence on FPP material parameters, such as the material attenuation factor, as well as on viscosity or sedimentation [12]. This means that a compromise must be found between the size and content of particles and the effect on the mechanical behaviour in materials designed for additive manufacturing by FPP methods. Nanoparticles will increase the mechanical properties in a more relevant way, but at the same time they will further increase the attenuation and reflection of the light due to the larger specific surface. As a result, it can be almost impossible to cure the polymeric layer required to obtain a successful printing process. Regarding the sedimentation of the reinforcement phase, smaller particles will be more easily stabilized and those larger will be less stable. Therefore, all these factors must be taken into account to analyse the viability and assessment of this approach, as well as the particle selection. Moreover, it should be considered that the increase of the solid load surface will also increase the scattering of the light decreasing the resolution of the MIP-SL printing equipment.

On the other hand, the evaluation of anisotropy in MIP-SL parts has been analysed in recent years [13,14,15,16]. One of the causes of anisotropy can be the pixilation of light emission system, which can produce a non-uniform conversion ratio distribution on the manufacturing plane [13,17]. This effect can be reduced or even removed through a post-curing process as a consequence of a homogenization of the conversion ratio distribution.

In this paper, alumina particles around the micron size have been used as a reinforcement in a commercial photocurable resin. The main objective of this paper is the analysis of stress-strain behaviour of compression samples manufactured by means of MIP-SL technology according to the solid load content of the reinforcement and orientation at the construction platform. Moreover, a FPP numerical model will be used to determine and optimize the energy exposure dose of each layer during the printing process to reach a full conversion ratio distribution along the layer thickness. Thus, the comparison between samples will be done for the same conversion ratio values and a properly assessment of the influence of reinforcement fillers will be done. Finally, the possibility to obtain similar mechanical properties using different layer thickness will be evaluated.

## 2. Materials and Methods

### 2.1. Formulation of Reinforced Photocurable Materials

A commercially available SPOT-HT resin (SpotA Materials, Barcelona, Spain) was used as a base photocurable resin and as a material matrix for reinforced developed materials. This base resin presents a compression secant modulus of 214 MPa (Section 3.3) for a specimen printed in the MIP-SL equipment used in this paper without a post-curing UV treatment. It also presents a low shrinkage value (1.5%) after its solidification. The alumina particles used as reinforcement (Aluminio óxido EssentQ^®^, Sharlab S.A., Barcelona, Spain) have an average particle size of ~2.5 µm, while the specific area is 1.12 m^2^/g. DISPERBYK-2013 (BYK, Wesel, Germany) was used as a dispersant in this work and BYK-1794 (BYK) as antifoam for the final formulation.

The reinforced photocurable materials were prepared by ball milling of the base resin, alumina particles and dispersant in a horizontal ball mill at 70 rpm for at least 48 h. Alumina was added into the resin with a solid load content in weight of 5%, 10% and 15%, respectively. Different dispersant ratios, in a range of 0.5 wt% to 5 wt% respect of the solid loading were prepared in order to optimize the dispersion stability of alumina. Dispersant content was determined by viscosity measurements of concentrated suspensions (70 wt% of alumina) in order to increase the variations in viscosity through dispersant content. Antifoam was added at 0.4 wt% in the final formulation after the ball milling, according to the producer recommendations.

Viscosity of the samples were performed in a stress-controlled rheometer (Haake Mars III, Thermo Fisher, Waltham, MA, USA) at 25 °C. A 35 mm plate-plate geometry was used with 0.5 mm gap. Viscosity curves were obtained from 0.1 to 200 s^−1^ of shear rate, in logarithmic increase, in CSR mode.

The stability of the suspension and the sedimentation rate have been evaluated by adding the suspensions in a graduated tube left at rest, avoiding contact with light at room temperature. The sedimentation rate has been determined by measuring the sedimentation front with respect to the maximum height of the resin.

The reactivities of the base and reinforced resin have been determined by cure depth tests. The tests have been carried out exposing small squares projected with the same light source as the MIP-SL equipment at a known distance of the resin surface. The surface of the resin was covered with a glass slide and the cured resin specimen grew top-down. Multiple samples were obtained at different energy exposure dose. The thicknesses of the specimens were measured with an indicator.

### 2.2. Analytical Model Based on a Frontal Photopolymerization Process

A discrete numerical model was used to determine the spatial-temporal monomer-to-polymer conversion (*χ*) according to the printing parameters and the material characteristics. The numerical model used was presented in [5] and it was based on the photo invariant numerical models of a Frontal Photopolymerization Process developed in [3,6,7]. The discrete numerical model considers the following assumptions: (i) the photopolymerization is only spread along the manufacturing direction, (ii) the energy exposure dose is constant for each pixel domain and (iii) the conversion process transmission along the construction plane (X-Y) is neglected. Consequently, the use of this numerical model does not take the scattering effects as a consequence of the addition of particles into account. More details about the discrete numerical model can be found in [5].

**Figure 1:**
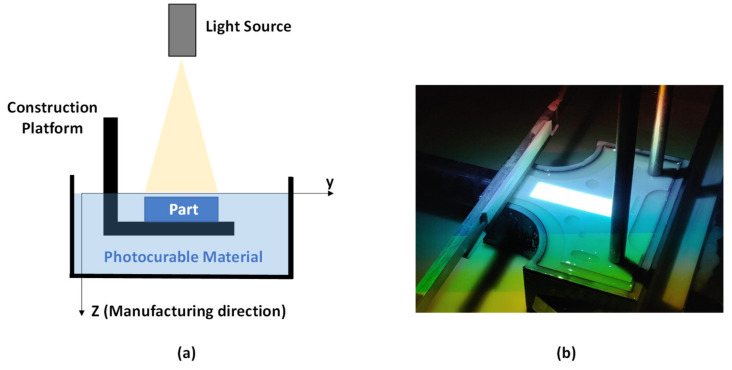
(**a**) Schematics of top-down MIP-SL printer. X and Y axis are located on the projection plane, whereas Z axis correspond to the manufacturing direction. (**b**) Detail of the construction platform of the MIP-SL equipment.

The conversion ratio of a photocurable material can be described by means of a dimensionless parameter (*Φ* *= χ/χ_max_*) through Equation (1), where *z* is the manufacturing direction (Figure 1), *μ* the material attenuation factor, *K* the material effective conversion rate and *d* the light exposure dose considering the whole manufacturing process.

(1) $$\Phi =1-exp\left(-K\cdot d\cdot exp\left(-\mu \cdot z\right)\right)$$

When the conversion ratio is higher than a threshold value (*Φ_c_*) the material is solidified. Furthermore, it is known that main mechanical properties of cured photocurable materials depend on its conversion ratio value [3,4]. Therefore, the use of numerical models to predict the material conversion ratio can be used to optimize the values of main printing parameters in order to enhance the material mechanical properties and to ensure a successful printing process.

The material parameters of FPP model have been experimentally obtained following the calibration procedure defined in [4,5]. The results can be found in Section 3.1.

During a MIP-SL printing process the same exposure dose is commonly used for each mask image projected. Therefore, *n* layer receives the energy dose (*d*_0_) from *n* mask image projected as well as an additional dose (*Δd_0,k_*) for each of the following *k* layers (*k* > *n*). Consequently, the total additional dose of *n* layer (*Δd_n_*) can be calculated through Equation (2).

(2) $$\Delta d_{n}=\overset{i=P}{\underset{i=k}{\sum }}\Delta d_{0,i}=\overset{i=P}{\underset{i=k}{\sum }}d_{0,i}\left(-\mu \left(i-n\right)\Delta z_{Layer}\right)$$

It is known that the total energy dose received in a specific location in the manufacturing domain is not constant as a consequence of the material attenuation factor as well as the printing process planning (energy exposure dose for each mask projected, number of layers, layer thickness, etc.). Consequently, a non-uniform conversion ratio distribution may be obtained which could cause some non-desired effects (non-uniform mechanical properties, shrinkage, etc.).

Other research considers mass and thermal effects [18] and oxygen inhibitory effects [19] in analytical models. These effects have not been considered in this paper.

### 2.3. Specimens and Experimental Setup for Compression Tests

**Figure 2:**
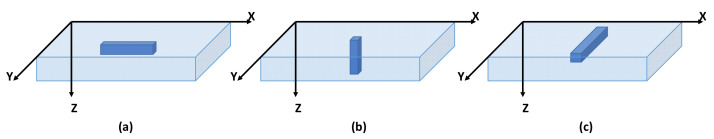
Orientation of compression samples in the printing manufacturing domain: (**a**) X, (**b**) Z and (**c**) Y.

Compression tests were done to obtain the mechanical behaviour (stress-strain curve) under an axial load for the four different types of reinforced photocurable materials described in Section 2.1 (0%, 5%, 10% and 15% of solid load content in weight of alumina particles). The specimens were manufactured in a top-down MIP-SL equipment (own building) (Figure 1). Initially, all samples were printed with a layer thickness of 75 μm and a temperature of 23 °C. Each specimen (12.7 × 12.7 × 50.4 mm) were manufactured at the same position on the construction platform to avoid its influence on the mechanical tests. The shape and dimensions of the coupons were set according to ASTM D 695-15 [20] for modulus measurements. A minimum of five samples were printed for each orientation X, Y and Z (Figure 2) in order to assess its influence on the material mechanical properties. As a result, a minimum of 60 compression specimens were tested.

**Figure 3:**
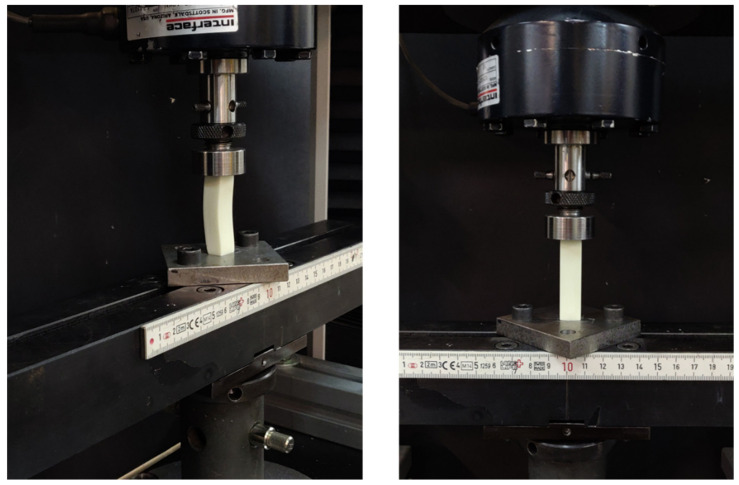
Experimental test set-up for reinforced compression specimens (12.7 × 12.7 × 50.4 mm).

Compression test were performed through a 3366 universal testing machine (Instron, Norwood, MA, USA) with a load capacity of 10kN (Figure 3). The loading rate were 2.6 mm/min for all samples. The strain was measured by means of the crosshead displacement of the testing machine. It is recommended to use crosshead displacement or the digital image correlation (DIC) technique to determine the specimen strain for materials with lower values of Young’s modulus [21].

The analysis of stress-strain behaviour was done through compression tests instead of tensile tests because of the smaller dimensions of the samples. Therefore, a lower volume of material was required to print all samples (top-down MIP-SL equipment), specially for samples with Z orientation.

## 3. Results and Discussion

### 3.1. Characteristics of Reinforced Materials

Each reinforced suspension needs to be stable a minimum amount of time to achieve a homogenous reinforcement distribution in the sample after the printing process; otherwise, the material characterization of printed parts will not give representatives values. In addition, the material viscosity is also a relevant factor to obtain a successful printing process through a MIP-SL equipment. Consequently, a properly selection and optimization of dispersants were done. To obtain the optimal dispersant through rheological tests, the minimum viscosity was sought for a given amount of dispersant between 0.5 wt% and 5 wt% relative to the solid to be dispersed. The dispersant optimization was performed at high solid concentrations (40 vol%) in order to increase the viscosity differences between measurements and to better control the added dispersant.

**Figure 4:**
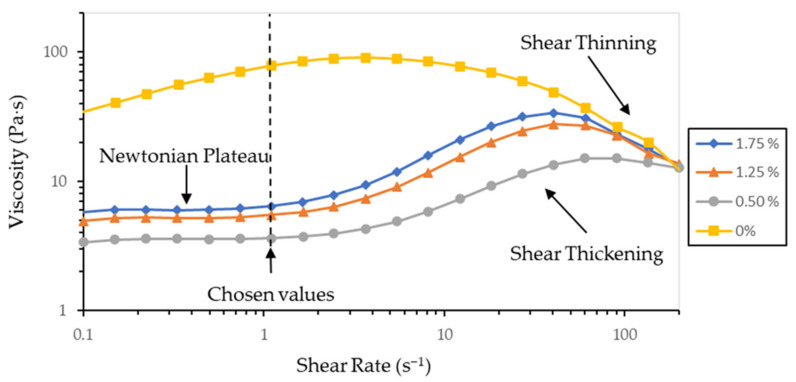
Viscosity curves for resin samples with 40 vol% alumina and different dispersant contents. It can be seen how the shear thickening and shear thinning behaviour shifts towards higher shear rates as it approaches to the optimum dispersant.

Figure 4 shows the evolution of the viscosity curves with the addition of dispersant. If no dispersant is added, the addition of the particles causes the reinforced resin to exhibit a dilatant or shear thickening behaviour from low to mid-shear rates, where it changes its behaviour to a pseudoplastic flow or shear thinning behaviour at high-shear rates. The addition of dispersant causes that at low shear rates there is a zone of Newtonian plateau that as the shear rate increases it transforms to a dilatant behaviour to change back to a pseudoplastic behaviour. As the formulation approaches to the optimum dispersant, the viscosity value in the Newtonian plateau is lower and the transition to dilating behaviour occurs at higher shear rates. To compare the viscosity values of all the obtained curves, the viscosity relative to the shear rate values of 1.1 s^−1^ belonging to the zone of the Newtonian plateau was chosen to facilitate their comparison.

**Figure 5:**
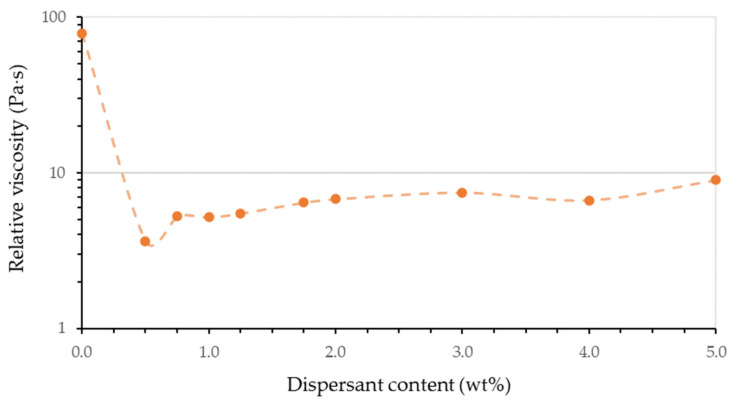
Evolution of relative viscosity at 1.1 s^−1^ of shear rate with the dispersant content at 40 vol% alumina resins. Each point corresponds to an average of at least three measured viscosity curves. It has a minimum viscosity of 0.5% dispersant indicating a better dispersion of the particles and the optimum dispersant chosen.

Figure 5 shows the evolution of the relative viscosity as a function of the dispersant content for a fixed solid content. As it can be seen, the addition of a small amount of dispersant greatly decreases the viscosity of the material. As the addition of more dispersant increases the viscosity of the resulting resin, it can be determined that the optimum dispersant is around 0.5 wt% with respect to the solid content.

Three different materials were formulated changing the solid content to obtain the reinforced suspensions (the same dispersant content was used in all of them, Table 1). The range of solid load content is compressed between 5 and 15 wt% since the addition of more reinforcing material usually leads to a worsening of the mechanical properties (Section 3.3).

**Figure 6:**
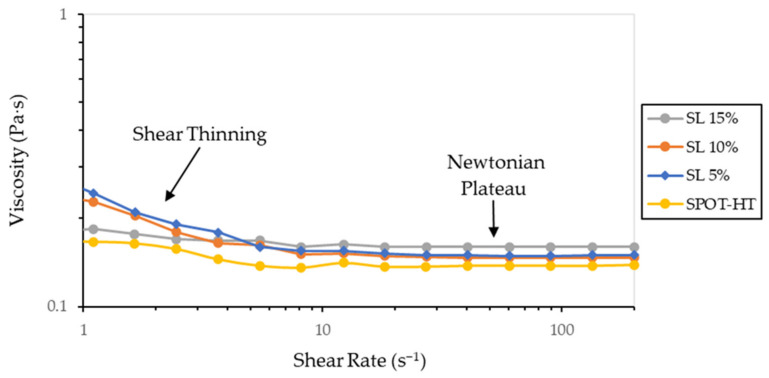
Comparison between selected viscosity curves of reinforced suspensions and base resin. Given the low viscosity of the material, it presents a first irregular shear thinning zone that varies slightly between measurements at low shear rates, but which stabilizes and always presents the same viscosity at high shear rates.

The prepared resins present viscosity curves that are presented in Figure 6. The developed materials show a very small increase in viscosity with respect to the base resin. Both the reinforced resins and the base resin show a mostly Newtonian behaviour except at low shear rates, where they show a small shear thinning behaviour. This behaviour so close to that of unreinforced resin will ensure that the material can be correctly used in commercial MIP-SL printers without problems.

**Figure 7:**
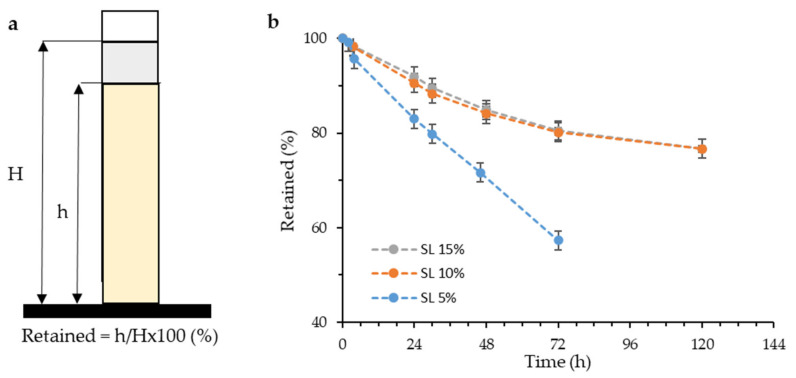
(**a**) Sedimentation rate calculation principle and (**b**) retained rate for reinforced resins indicating the sedimentation rate of the different developed materials.

Once the resin was formulated, the stability of the suspension was verified by means of sedimentation tests of the particles. A stable suspension is required to achieve a homogeneous impression with constant properties throughout the printed part. As the suspension is allowed to stand, the particles tend to settle due to their very high density and the low viscosity of the suspending medium. To measure the sedimentation rate, the percentage of retained material was calculated as can be seen in Figure 7a, where *H* is the height of the total resin and *h* is the height of the resin where there is still the initial particle concentration. The values of material retained as a function of time are shown in Figure 7b for each of the reinforced resins.

Materials that only have up to a maximum of 15 wt% solid have very low sedimentation rates compared to other similar materials [22]. There is a substantial difference between the settling rate of the material at 5 wt% and those at 10 and 15 wt%. This difference may be the cause of a minimum limit of solid that the dispersant can stabilize at rest, forming retaining structures. It is possible that at 5 wt% there is not enough solid surface for the dispersant to be able to structure, but if there is enough from 10 wt%, which would improve the capacity to retain the solid of the material in suspension, explaining this difference in the sedimentation rate.

**Figure 8:**
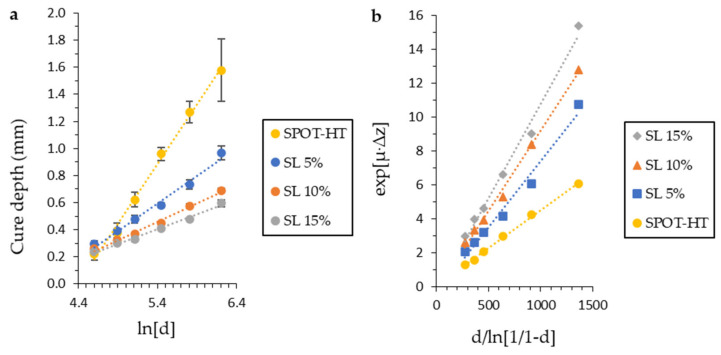
Evolution of reactivity behaviour front solid load incorporation. From the slopes of each line, (**a**) the *µ* and (**b**) *K* values are obtained for each formulation according to Equation (1).

The reactivity of the reinforced resins was characterized as established. From the cure depth data related to the exposure energy dose, the model parameters were calculated according to the Equation (1) and the plot of the graphs in Figure 8a,b. Each point for an energy concentration and dose has been determined by a minimum of five tests. The specific values for each determined constant of the model of Equation (1) can be seen in Table 2.

### 3.2. Definition of Printing Parameters

The FPP model presented is Section 2 has been used to determine the main printing parameters in order to obtain a conversion ratio distribution as homogenous as possible along the manufacturing direction. The following assumptions have been made: (i) all suspensions present the same maximum conversion value (χ_max_) and (ii) the same threshold conversion ratio (Φ_c_). Experimental measurements have been done to validate these assumptions by means of FTIR tests. Similar values for threshold conversion ratio and maximum conversion value (differences less than 5% between samples) have been obtained. Therefore, it has been assumed that the influence of solid load content on these parameters can be neglected. Obviously, this assumption cannot be extrapolated for higher solid load content values or different types of reinforcement fillers. Table 2 shows the FPP materials parameters for each material suspension. An increment of material attenuation factor (*μ*) and material effective conversion rate (*K*) has been obtained as a consequence of the increment of solid load content reinforcement. The change of these parameters cannot be neglected and it has to be considered for the optimization of exposure dose through the numerical model.

First of all, the exposure dose of each printed layer (*d*_0_) has been adjusted in order to obtain a full conversion ratio distribution (*Φ* > 0.995) along the whole layer thickness for each material suspension during the printing process (The conversion ratio is based on the maximum conversion *χ_max_* achievable for the MIP-SL light source). As a result, a similar mechanical behaviour (stress-strain curves) between samples printed in different directions (X, Y and Z) is expected to be achieved. A UV post-curing process has no been applied to any specimen.

The exposure energy dose defined for each material suspension is also shown in Table 2 in order to obtain a full conversion ratio for each case for a layer thickness of 75 μm. Higher values of energy doses are obtained for the material suspensions with higher solid load content as a consequence of the increment of the material attenuation factor. Therefore, the printing speed for reinforced materials is significantly lower. It is important to point out that an uncontrolled increment of the material attenuation factor may reduce the cure depth reducing the material printability. Thus, its value needs to be considered and controlled when a new reinforced suspension is developed.

### 3.3. Analysis of Mechanical Properties

Sixty different samples (five samples (S1–S5) for each solid load content and printing orientation (X, Y and Z)) have been performed by means of compression tests (defined in Section 2.3) in order to evaluate the stress-strain curve relationship according to the printing direction as well as the solid load content.

**Figure 9:**
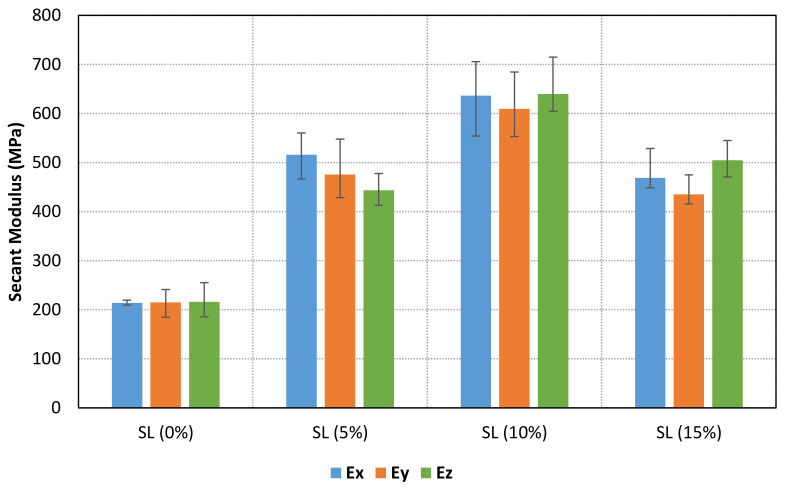
Secant modulus mean values for each printing orientation (X in blue, Y in orange and Z in green) according to the solid load content. It can be observed an enhancement of secant modulus after the addition of reinforcement particles.

**Figure 10:**
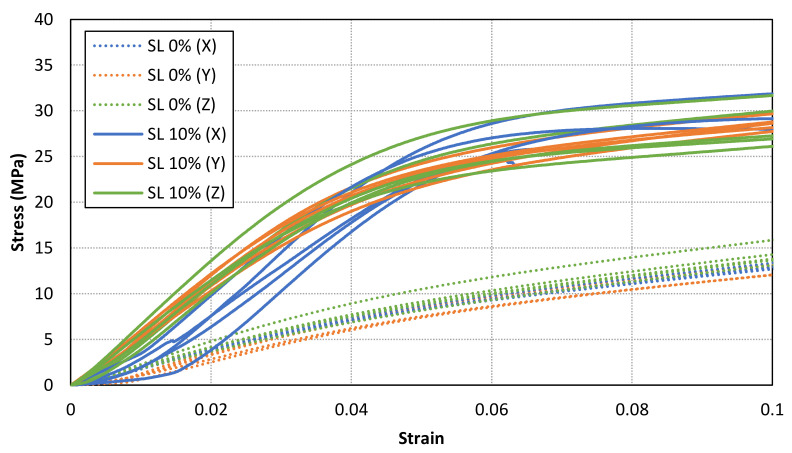
Comparison of stress-strain curves for all tested specimens with a solid load content of 0% and 10%. Similar stress-strain curves are found for different orientation samples with the same solid load content.

A material behaviour with no clear Hookean region has been found for all samples. Therefore, a secant modulus (0.6% of longitudinal strain after toe region compensation) for each specimen has been calculated. The results are shown in Table 3, Table 4 and Table 5 for X, Y and Z samples, respectively. Furthermore, the mean values for each case are compared in Figure 9. The results show an increment of secant modulus after the addition of fillers in the base photocurable material. Furthermore, the enhancement of mechanical properties is not lineal nor constant to the increment of solid load content. In fact, the maximum values are achieved for a solid load content of 10%, whereas lower mechanical properties are obtained for a higher solid load content (15%). In addition, the stress-strain curves for all samples with a solid load content of 0% and 10% are compared in Figure 10. Consequently, an optimal solid load content can be found to increase mechanical properties.

**Figure 11:**
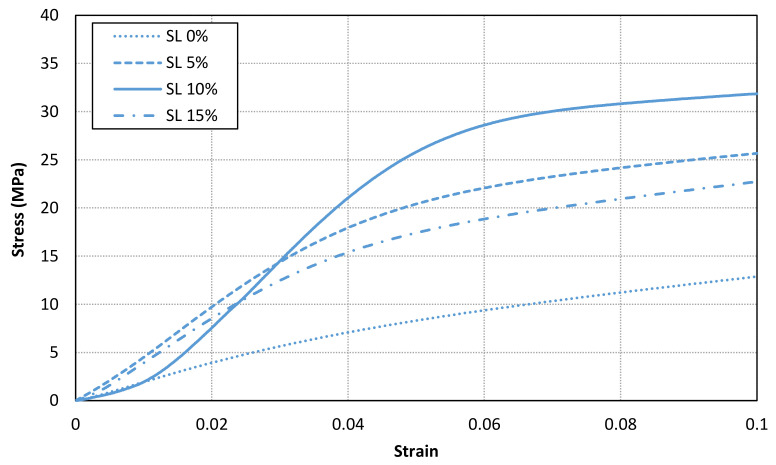
Stress-strain curves for a representative compression sample for each solid load content for X orientation.

**Figure 12:**
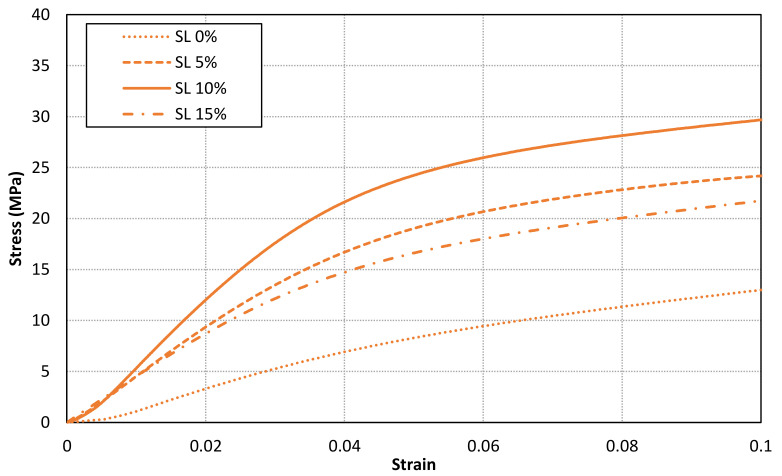
Stress-strain curves for a representative compression sample for each solid load content for Y orientation.

**Figure 13:**
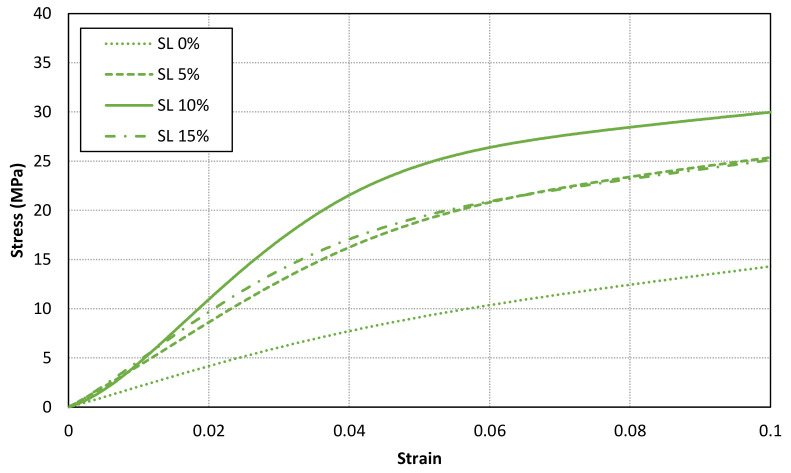
Stress-strain curves for a representative compression sample for each solid load content for Z orientation.

**Figure 14:**
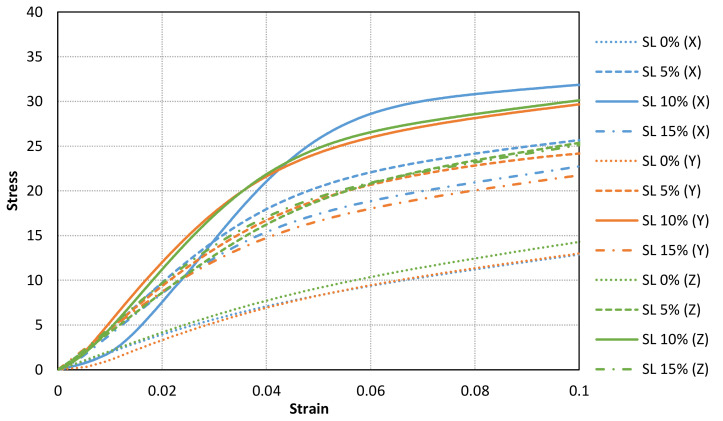
Comparison of stress-strain curves between different solid load content and printing orientation (Blue lines for X samples, oranges lines for Y samples and green lines for Z samples).

Figure 11, Figure 12 and Figure 13 shows the stress-strain curves for a representative compression sample for each printing orientation (X, Y and Z). It can be observed the enhancement of mechanical properties after the addition of reinforcement fillers. In addition, the stress-strain curves for each orientation specimens and solid load content are compared in Figure 14. Similar stress-strain relationships are obtained for each orientation for the same solid load content. In fact, the results do not present a significantly difference between stress-strain curves of each orientation (Figure 10 and Figure 14). Consequently, it seems reasonable to consider an isotropic material behaviour on MIP-SL printed parts if a full conversion ratio is achieved during the printing process. This assumption can also be done after a post-curing process [17]. Thus, achieving a uniform conversion ratio on the printed part domain is a key factor to achieve an isotropic material behaviour.

The values of secant modulus also show a reasonably low standard deviation. It is important to point out that each specimen was printed at the same location on the manufacturing domain (one sample for each printing).

### 3.4. Optimization of Printing Parameters According to the Layer Thickness

The influence of main printing parameters (layer thickness and exposure energy dose) has been analysed in this section. On one hand, the use of thinner layer thickness allows a higher resolution and accuracy along the manufacturing direction. Furthermore, it can be easy to obtain a valid printing process ensuring a properly bonding between layers, especially with photocurable materials with a high attenuation factor value.

On the other hand, a thicker layer thickness can be defined in order to increase the manufacturing speed. Nevertheless, several issues need to be taken into account. First of all, a higher gradient of conversion ratio (non-uniform distribution) is expected along the layer thickness. As a result, non-uniform and/or lower mechanical properties could be obtained. In addition, the exposure energy dose must be increased to reach a full conversion ratio (as uniform as possible) along the manufacturing direction.

Two additional set of compression samples with Y orientation and a reinforcement solid load content of 10% have been printed with a thicker layer thickness (150 μm instead of 75 μm). Each set has been printed with a different energy exposure dose. The first one, with the same energy exposure dose used for previous samples (252 mJ/cm^2^). On the contrary, the analytical model described in Section 2.2 has been used to determine the required energy exposure dose (580 mJ/cm^2^) to obtain a full conversion ratio.

**Figure 15:**
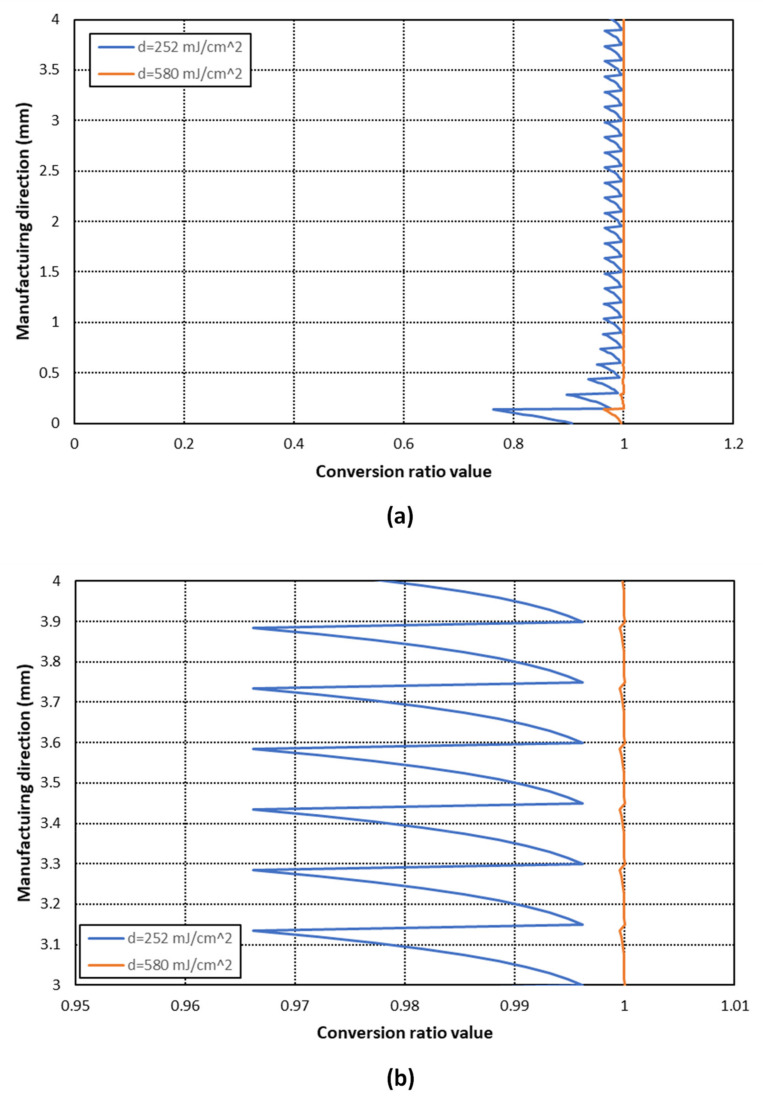
Comparison of conversion ratio distribution along the manufacturing direction for the photocurable material with a 10% SL and a layer thickness of 150 μm according to the energy exposure dose. (**a**) It can be observed that a large difference is found in the last printed layers (**b**) Detail of conversion ratio values excluding the last printed layers.

The comparison of conversion ratio distribution along the manufacturing direction between each case can be seen in Figure 15. The results show that a practically full and uniform conversion (Φ > 0.995) is achieved with an energy exposure dose of 580 mJ/cm^2^, whereas a clearly non-uniform distribution is obtained with an exposure dose of 252 mJ/cm^2^. This effect is more notorious in the last printed layers. Therefore, the energy exposure dose needs to be calculated according to the layer thickness and material parameters in order to achieve a higher and more uniform conversion ratio distribution.

**Figure 16:**
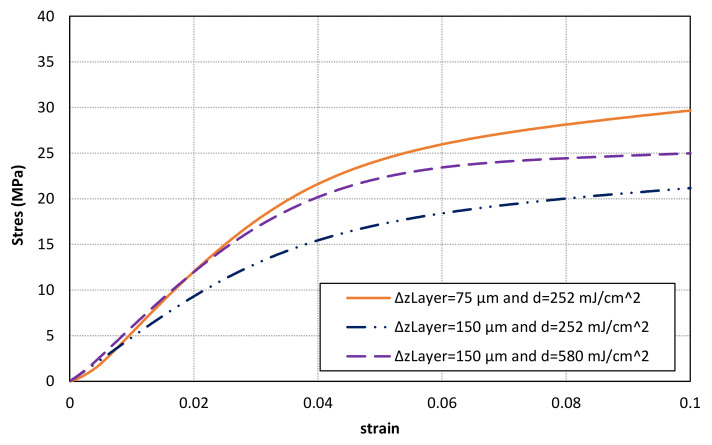
Comparison of stress-strain curves for the photocurable material SL10% according to the printing parameters.

The results of compression tests can be found in Table 6 and Figure 16. A similar secant modulus is obtained for samples with layer thickness of 75 μm and 150 μm after adjusting the energy exposure dose. On the contrary, lower values are achieved for samples with thicker layers and non-optimized energy exposure dose. Thus, a properly definition of printing parameters is a key factor to increase the conversion ratio value and, consequently, to enhance mechanical properties. However, the stress-strain curves of samples with thicker layers seem to have lower stress values after the initial part although similar secant modulus have been obtained. Finally, it can also be observed that a clearly lower stress-strain relationship is found for samples without a full conversion distribution. Thus, it is recommended to analyse and optimize the printing parameters in order to achieve a full conversion distribution and, consequently, to enhance stress-strain material behaviour.

## 4. Conclusions

The paper has presented an analysis of the stress-strain behaviour of compression samples manufactured by means of Mask Image Projection based on Stereolithography technology. First of all, the results show the influence of reinforcement particles on the material FPP parameters. The increase of material attenuation factor has a relevant influence on the required exposure energy dose to reach a full conversion ratio distribution along the layer thickness, which is a key factor to enhance the main mechanical properties. Consequently, the use of analytical and numerical models to determine the exposure dose is clearly recommended to optimize and improve the mechanical behaviour of printed parts. At the same time, a stable resin has been obtained allowing the manufacturing of homogenous parts. This is a key factor for a correct evaluation of the mechanical properties once the labels of the new materials have been printed. The particle size chosen for the formulation of the resin seems adequate since it achieves the improvement of properties and a stable suspension, at the same time that it does not compromise the photocurable properties of the resin. The amount of solid added to the material as reinforcement can be defined as the appropriate to observe the entire area interesting in terms of effect on mechanical properties.

The results show a relevant enhancement of stress-strain behaviour of reinforced materials under a compression load. Nevertheless, mechanical properties seem to decrease after reaching a certain value of solid load content. Consequently, the solid load content of reinforcement fillers needs to be optimized in order to obtain better stress-strain material behaviour. The optimal particle size for this formulation with the chosen alumina size is around 10 wt%. The limit is somewhat higher than that found in other studies using alumina particles of a smaller size [11].

In addition, a similar secant elastic modulus has been experimentally measured for full converted compression samples printed with different orientations. This phenomenon has been practically obtained for the whole range of solid load content analysed. Consequently, it seems possible to considerer an isotropic material behaviour of printed parts when a full conversion ratio is achieved during the printing process. Therefore, the use of numerical, analytical or experimental models are required to determine the conversion ratio distribution according to the printing process characteristics. However, this effect cannot be directly extrapolated to other types of reinforcement or conversion ratio distribution before a further research.

Finally, similar secant modulus have been obtained for specimens manufactured with different layer thickness by adjusting the exposure energy dose. Therefore, the use of numerical models is also recommended to estimate the optimal exposure time according to the layer thickness defined during the printing process.

## Figures and Tables

**Table 1:** Reinforced resins formulation.

**Code**	**SPOT-HT (wt%)**	**Alumina (wt%)**	**Dispersant (wt% ^1^)**	**Antifoam (wt%)**
SL 5%	94.6	5.0	0.5	0.4
SL 10%	89.6	10.0	0.5	0.4
SL 15%	84.6	14.9	0.5	0.4

^1^ Respect to the solid content.

**Table 2:** Material FPP parameters and exposure energy dose required to achieve a full conversion ratio during the printing process according to the reinforcement solid load content.

	SL 0%	SL 5%	SL 10%	SL 15%
*μ* (mm^−1^)	1.139	2.45	3.69	4.59
*K*(cm^2^/mJ)	0.0045	0.0073	0.0094	0.0107
*d*_0_ (mJ/cm^2^)	126	227	252	277

**Table 3:** Secant modulus (MPa) for X samples.

E_X_ (MPa)							
**SL**	**S1**	**S2**	**S3**	**S4**	**S5**	**Mean**	**St**
0%	219.48	210.42	217.91	209.29	212.61	213.94	4.53
5%	540.18	466.34	560.42	519.42	493.51	515.97	37.22
10%	705.37	674.87	553.99	586.38	661.76	636.47	63.59
15%	528.59	448.65	461.06	454.79	450.21	468.66	33.85

**Table 4:** Secant modulus (MPa) for Y samples.

**E_Y_ (MPa)**							
**SL**	**S1**	**S2**	**S3**	**S4**	**S5**	**Mean**	**St**
0%	241.00	229.69	223.51	184.60	196.89	215.14	23.54
5%	458.95	495.90	547.48	428.32	447.81	475.69	47.07
10%	684.78	552.60	644.77	576.72	626.97	609.41	53.00
15%	474.98	473.70	420.99	415.50	453.48	434.91	28.30

**Table 5:** Secant modulus (MPa) for Z samples.

**E_Z_ (MPa)**							
**SL**	**S1**	**S2**	**S3**	**S4**	**S5**	**Mean**	**St**
0%	255.02	213.95	185.61	211.49	212.41	215.70	24.92
5%	441.14	442.05	443.19	477.44	412.45	443.25	23.04
10%	650.36	604.43	714.72	607.68	621.36	639.71	45.68
15%	504.41	470.35	488.02	516.63	544.94	504.87	28.37

**Table 6:** Comparison between Secant modulus (MPa) of SL10% Y samples.

E_Z_ (MPa)								
Δz_Layer_ (μm)	d (mJ/cm^2^)	S1	S2	S3	S4	S5	Mean	St
75	252	684.78	552.60	644.77	576.72	626.97	609.41	53.00
150	252	450.20	485.75	464.27	507.19	450.66	471.61	24.58
150	580	558.73	635.36	597.11	605.00	614.69	602.18	28.19

## Data Availability

The data is available from the author upon request.
